# Effects of Motion in Ultrashort Echo Time Quantitative Susceptibility Mapping for Musculoskeletal Imaging

**DOI:** 10.3390/jimaging11100347

**Published:** 2025-10-06

**Authors:** Sam Sedaghat, Jinil Park, Eddie Fu, Fang Liu, Youngkyoo Jung, Hyungseok Jang

**Affiliations:** 1 Department of Diagnostic and Interventional Radiology, University Hospital Heidelberg, 69120 Heidelberg, Germany; samsedaghat1@gmail.com; 2 Department of Radiology, University of California, Davis, Sacramento, CA 95817, USA; jiipark@health.ucdavis.edu (J.P.); eyjfu@health.ucdavis.edu (E.F.); yojung@health.ucdavis.edu (Y.J.); 3 Athinoula A. Martinos Center for Biomedical Imaging, Harvard Medical School, Charlestown, MA 02129, USA; fliu12@mgh.harvard.edu; 4 Department of Radiology, Massachusetts General Hospital, Boston, MA 02114, USA

**Keywords:** ultrashort echo time (UTE), quantitative susceptibility mapping (QSM), motion artifacts, musculoskeletal (MSK) imaging, magnetic resonance imaging (MRI)

## Abstract

Quantitative susceptibility mapping (QSM) is a powerful magnetic resonance imaging (MRI) technique for assessing tissue composition in the human body. For imaging short-T2 tissues in the musculoskeletal (MSK) system, ultrashort echo time (UTE) imaging plays a key role. However, UTE-based QSM (UTE-QSM) often involves repeated acquisitions, making it vulnerable to inter-scan motion. In this study, we investigate the effects of motion on UTE-QSM and introduce strategies to reduce motion-induced artifacts. Eight healthy male volunteers underwent UTE-QSM imaging of the knee joint, while an additional seven participated in imaging of the ankle joint. UTE-QSM was conducted using multiple echo acquisitions, including both UTE and gradient-recalled echoes, and processed using the iterative decomposition of water and fat with echo asymmetry and least-squares estimation (IDEAL) and morphology-enabled dipole inversion (MEDI) algorithms. To assess the impact of motion, datasets were reconstructed both with and without motion correction. Furthermore, we evaluated a two-step UTE-QSM approach that incorporates tissue boundary information. This method applies edge detection, excludes pixels near detected edges, and performs a two-step QSM reconstruction to reduce motion-induced streaking artifacts. In participants exhibiting substantial inter-scan motion, prominent streaking artifacts were evident. Applying motion registration markedly reduced these artifacts in both knee and ankle UTE-QSM. Additionally, the two-step UTE-QSM approach, which integrates tissue boundary information, further enhanced image quality by mitigating residual streaking artifacts. These results indicate that motion-induced errors near tissue boundaries play a key role in generating streaking artifacts in UTE-QSM. Inter-scan motion poses a fundamental challenge in UTE-QSM due to the need for multiple acquisitions. However, applying motion registration along with a two-step QSM approach that excludes tissue boundaries can effectively suppress motion-induced streaking artifacts, thereby improving the accuracy of musculoskeletal tissue characterization.

## 1. Introduction

Magnetic susceptibility is a fundamental physical property of tissue that describes its response to an external magnetic field [1,2]. This property varies across tissues due to differences in their chemical composition and structure. Since MRI relies on a strong magnetic field for imaging, it is inherently influenced by tissue susceptibility. Most biological tissues are mildly diamagnetic, meaning they generate a local dipole field that slightly opposes and reduces the external magnetic field. In contrast, certain substances, such as hemosiderin and deoxygenated hemoglobin in red blood cells, are paramagnetic, which enhances the local magnetic field within the MRI scanner.

Quantifying magnetic susceptibility in tissues offers valuable insight into disease processes, as it reflects underlying chemical composition changes that may indicate pathology. For instance, elevated susceptibility in the liver is often linked to iron deposition, a potential biomarker for liver disease [3]. Similarly, increased susceptibility in the brain may signal iron accumulation, which has been associated with neurological conditions such as Alzheimer disease and multiple sclerosis [4,5,6]. Accumulations of hemosiderin or microhemorrhages can also increase local susceptibility. Conversely, decreased susceptibility may result from tissue calcification, an essential marker in cancer evaluation [7].

Imaging of the musculoskeletal (MSK) system, a significant field in radiology, is essential for evaluating joints, cartilage, tendons, ligaments, and bone. MSK imaging plays a crucial role in diagnosing various diseases, including trauma, degenerative conditions, and inflammatory conditions, thereby informing therapeutic decision-making. Quantitative susceptibility mapping (QSM) has been investigated as a promising MRI technique for characterizing the biochemical composition of tissues in the human body [8,9,10,11,12]. To apply QSM to short T2 tissues in the MSK system, ultrashort echo time (UTE) imaging can be beneficial, as it improves sensitivity to signals from essential tissues, such as tendons, ligaments, menisci, osteochondral junctions, and bone [13,14,15,16,17]. Data acquisition for UTE-based QSM (UTE-QSM) typically involves multiple repeated scans to achieve short echo spacing between UTE images. The multiple scans may cause unwanted motion between scans, known as inter-scan motion, leading to misregistration between images. This effect can adversely affect the accuracy of UTE-QSM due to streaking artifacts caused by motion-contaminated pixel-wise phase evolution, which becomes more critical near tissue boundaries. While motion registration can be beneficial, achieving perfect motion registration with images at different TEs with various T2-weighting and chemical shift artifacts is challenging.

In this study, we investigate the effect of motion in UTE-QSM. We hypothesized that an error of susceptibility may occur near the tissue interface, due to the misregistration of the pixels between different echo times (TEs) acquired in the repeated scans. The error in the tissue boundary can be propagated to the entire susceptibility map. Therefore, it is essential to understand the impact of motion and explore potential solutions to address this issue. With the in vivo experiment conducted on volunteers, we demonstrate the effect of inter-scan motion and the efficacy of methods to reduce motion-induced artifacts in UTE-QSM, including conventional rigid-body affine registration and a new approach that considers tissue boundaries.

## 2. Materials and Methods

### 2.1. Study Participants

All experiments were performed in accordance with relevant guidelines and regulations. All experimental protocols were approved by the Institutional Review Board of the University of California, San Diego. Informed consent was obtained from all subjects and/or their legal guardian(s). A group of healthy volunteers were recruited for this study, and all participants underwent either an MRI of the knee or ankle, with approval from the institutional review boards.

### 2.2. UTE Sequence

**Figure 1:**
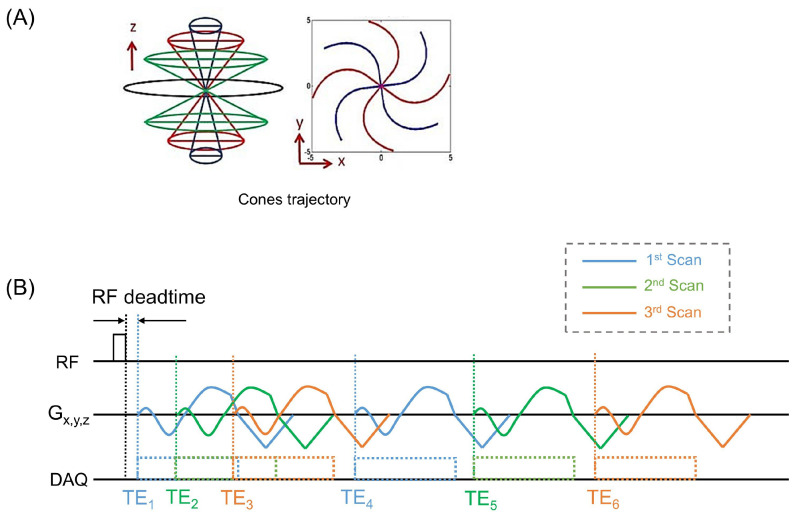
MRI pulse sequence for UTE-QSM. (**A**) 3D cones k-space trajectory and (**B**) pulse sequence of interleaved dual echo 3D cones UTE imaging.

Conventional 3D radial UTE imaging sequences realize dramatically shortened TEs by utilizing half-projection imaging performed immediately after radiofrequency (RF) excitation and the subsequent RF coil dead time (a blind time due to the switching time from RF transmit mode to receive mode). The half-projection imaging is conducted based on a readout gradient with an omitted dephasing gradient, resulting in center-out radial k-space trajectories with varying polar and azimuthal angles to encode 3D spherical k-space. The radial trajectories can be twisted to cover the k-space more efficiently, which is known as 3D cones UTE imaging (Figure 1A) [18].

In this study, a dual-echo UTE sequence utilizing a 3D cones trajectory was employed (Figure 1A). To obtain six input images for UTE-QSM, three interleaved scans with varying echo times were performed (Figure 1B). Interleaved acquisition is essential for UTE-QSM, as it enables the short echo spacing required to capture rapidly decaying signals from musculoskeletal tissues such as bone, tendon, ligament, and meniscus.

### 2.3. MRI Protocol

The study participants underwent MRI in a 3T clinical MRI scanner (GE Healthcare, Chicago, IL, USA) using the following imaging parameters: (1) Knee UTE-QSM: 8-channel transmit/receive knee coil, flip angle = 10°, repetition time = 10 ms, three dual echo scans with TE = 0.032/2.7 ms, 0.2/3.7 ms, and 0.6/4.7 ms, FOV = 150 × 150 × 86.4 mm^3^, matrix size = 220 × 220 × 96, and scan time = 17 min 16 s; (2) Ankle UTE-QSM: 8-channel receive-only ankle coil, and parameters matched with knee UTE-QSM.

### 2.4. UTE-QSM Process

Unlike the QSM of the brain, the fat signal should be considered for the QSM in the MSK system. The protons in fat exhibit a chemical shift of around −3.5 ppm away from the water protons, where the resonance frequency is lower than in the water protons. This chemical shift can be a significant source of error in QSM, as it utilizes information about off-resonance phase evolution to estimate magnetic susceptibility. Therefore, in the study, the UTE-QSM was performed based on fat-water signal fitting using iterative decomposition of water and fat with echo asymmetry and least-squares estimation (IDEAL) [19]. The IDEAL produces a B0 field map by fitting the signal acquired at different TEs, considering both T2* decay and fat chemical shift. The acquired complex signal is fitted to the following model in the gradient echo at a TE of *t*.

(1) $$S\left(t\right)=\left(S_{W}e^{-\frac{t}{T2^{*}}}+S_{F}\sum_{k=1}^{N}\alpha_{k}e^{i2\pi f_{k}t}\right)e^{i(\phi_{0}+2\pi f_{B0}t)},$$

where *S_W_* and *S_F_* are magnitudes of water and fat signals at the zero TE, T2* denotes an effective spin-spin relaxation time, $\alpha_{k}$ and $f_{k}$ are the relative amplitude and chemical shift frequencies of multiple peaks in the fat spectrum (*N* major peaks), and $\phi_{0}$ and $f_{B0}$ denote the initial phase offset and off-resonance frequency due to B0 inhomogeneity, respectively.

The acquired B0 field map (i.e., $f_{B0}$) was subsequently processed using projection onto dipole fields (PDF) to remove the background field and obtain a tissue-induced local field map [20]. Finally, the local field map was processed using morphology-enabled dipole inversion (MEDI) algorithms [21] to produce a susceptibility map.

### 2.5. Two-Step UTE-QSM Using Tissue Boundary Information

We implemented a two-step UTE-QSM method to identify the source of motion-related artifacts and developed a new approach to mitigate them in UTE-QSM. This method is based on the hypothesis that pixelwise misregistration is particularly problematic near tissue boundaries, where adjacent tissues exhibit markedly different properties, such as T2* relaxation times, susceptibility values, and chemical shifts. In such regions, misalignment of pixels between echo times can introduce significant errors in signal fitting due to deviations in the complex signal, including both magnitude and phase components.

**Figure 2:**
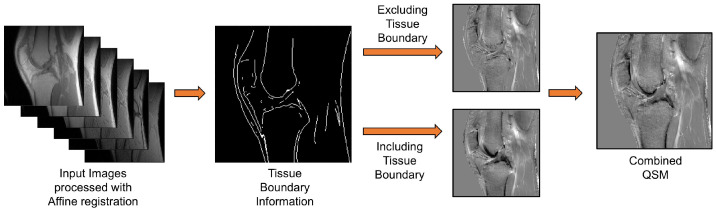
The two-step UTE-QSM utilizes tissue boundary information to mitigate the adverse effects of inter-scan motion near tissue boundaries, assuming that significant errors arise from uncorrected motion in this region. The images are first affine-registered and then processed using two different masking schemes (including or excluding tissue boundaries). After the QSM process, the estimated susceptibility maps are merged.

Figure 2 presents the framework of the proposed two-step UTE-QSM approach. First, complex images acquired at different echo times are aligned using a rigid body affine registration. Next, edge detection is performed on a UTE image to generate a line edge map [22]. Pixels within a 2-pixel radius of the detected edges are then excluded from the initial QSM reconstruction to minimize streaking artifacts arising from residual motion near tissue boundaries. A second QSM reconstruction is subsequently performed using the full dataset. Finally, the two susceptibility maps are combined using alpha blending to produce the final output.

### 2.6. Data Processing

All data processing was conducted using Matlab 2024a (The MathWorks, Inc., Natick, MA, USA). First, all images were motion-registered using the UTE images at the first TE (32 µs) as a fixed image (a reference image), utilizing an affine transformation-based rigid registration algorithm. The UTE-QSM was performed using a homemade code based on the fat-water toolkit [23] and the MEDI toolkit [21]. The QSM process used a regularization parameter of 1000. In the two-step UTE-QSM approach, a 3D Canny edge detection algorithm with a threshold of 0.3 was applied to images at the final echo time (4.7 ms). The resulting edge map was then refined using a 3D morphological dilation algorithm with a spherical structural element of 2-pixel radius to incorporate neighboring pixels. After performing the QSM process with and without including the detected tissue boundaries, the two susceptibility maps were merged using alpha blending based on a Gaussian filter (sigma = 1 pixel).

Estimated susceptibility values were evaluated in several regions of interest (ROIs) within the knee and ankle joints, including the patellar tendon, posterior cruciate ligament (PCL), meniscus, and muscle in the knee, as well as the Achilles tendon and muscle in the ankle. A Student’s *t*-test was conducted to compare susceptibility values across three approaches: UTE-QSM without registration, UTE-QSM with affine registration, and the two-step UTE-QSM approach. A *p*-value of less than 0.05 was considered statistically significant.

## 3. Results

### 3.1. Baseline Data

Eight healthy male volunteers (31.9 ± 4.9 years) underwent knee joint UTE-QSM, while another seven healthy male volunteers (28.4 ± 2.9 years) participated in ankle joint UTE-QSM. Written informed consent was obtained from all participants before the MRI.

### 3.2. UTE-QSM with IDEAL

**Figure 3:**
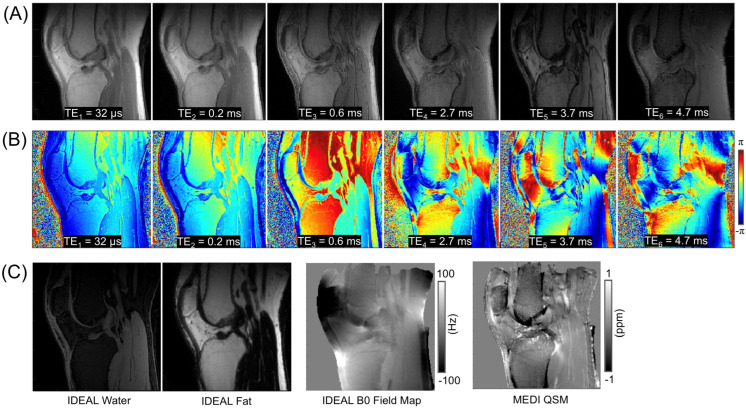
An example of UTE-QSM processing utilizing both Iterative Decomposition of water and fat with Echo Asymmetry and Least-squares estimation (IDEAL) and Morphology-Enabled Dipole Inversion (MEDI) algorithms. (**A**) Magnitude images and (**B**) phase images at six different TEs, and (**C**) results from IDEAL and MEDI.

An example from a representative subject (a 36-year-old male) is shown in Figure 3. Figure 3A,B display the magnitude and phase images obtained at six different TEs, along with the resultant field map from IDEAL and the susceptibility map from MEDI. Figure 3C shows the results from IDEAL (water and fat images, as well as the estimated B0 field map) and the final QSM results.

### 3.3. Effect of Motion and Motion Correction

**Figure 4:**
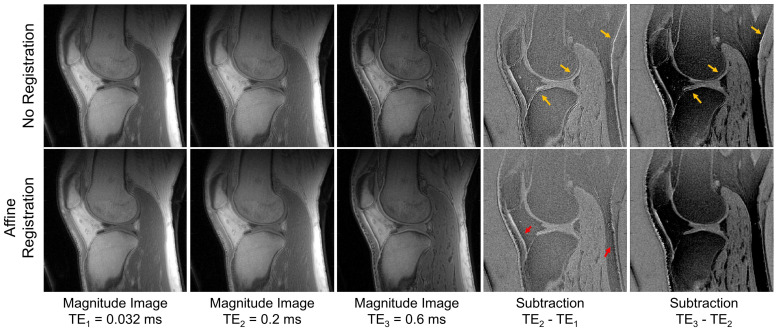
Motion correction using affine registration: The rigid body registration scheme based on affine transformation improved bulk motion in the knee joint, as evidenced by subtraction images between different echoes. Strong boundary artifacts caused by misregistration (indicated by yellow arrows) are significantly reduced after affine registration. However, a residual boundary effect remains due to uncorrected motion (indicated by red arrows).

Figure 4 illustrates the impact of inter-scan motion in interleaved multiple echo UTE imaging. The subtracted images reveal significant boundary effects (yellow arrows), which result from misaligned pixels caused by motion between acquisitions. When affine transformation-based motion registration is applied, these effects are notably reduced. However, a residual boundary effect persists due to uncorrected motion (indicated by red arrows).

### 3.4. UTE-QSM on the Knee and Ankle Joints

**Figure 5:**
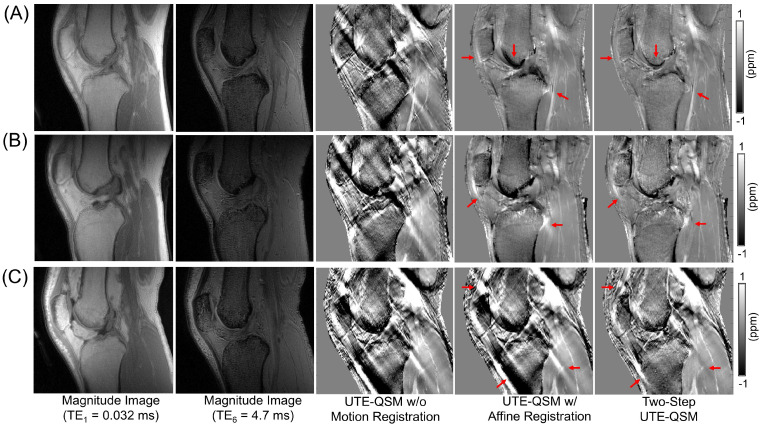
Knee UTE-QSM results from three representative healthy volunteers ((**A**), (**B**), and (**C**), aged 28, 36, and 31 years, respectively). Mild motion was observed in the top and middle subjects (**A**,**B**), while strong motion was noted in the bottom subject (**C**). Sole affine registration improved UTE-QSM results for subjects (**A**,**B**), but it did not reduce streaking artifacts in subject (**C**), which was affected by strong motion. In all cases, the two-step UTE-QSM effectively mitigated streaking artifacts in the tissues of interest (highlighted by red arrows). The two-step UTE-QSM notably improved the results for subject (**C**), despite the strong motion.

**Figure 6:**
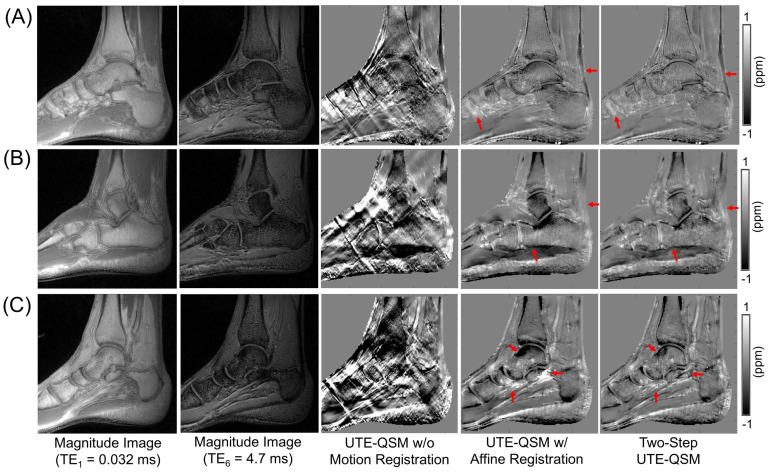
Ankle UTE-QSM results from three representative healthy volunteers ((**A**), (**B**), and (**C**), aged 32, 31, and 28 years, respectively). Affine registration notably improved the streaking artifacts. The two-step UTE-QSM further enhanced the depiction of short T2 tissues, such as the Achilles tendon and muscle, with dramatically reduced streaking artifacts (highlighted by red arrows).

Figure 5 and Figure 6 show the UTE-QSM results for the knee and ankle joints from six representative subjects. Affine transformation-based motion registration significantly improved the QSM results, although residual streaking artifacts remain due to uncorrected motion. Two-step UTE-QSM, incorporating tissue boundary information, further enhanced the results by reducing the remaining streaking effects (highlighted by red arrows). Table 1 presents the susceptibility values estimated in all ROIs using the three approaches. In the knee joints, significant differences in tissue susceptibility estimates are observed in most ROIs when two-step UTE-QSM is applied (*p*-values < 0.05). However, no significant changes in the ROIs were found in the ankle joints, particularly for the Achilles tendon, likely due to its greater rigidity, which is less affected by non-rigid motion.

## 4. Discussion

Unlike conventional T2* relaxometry, which is also sensitive to tissue magnetic susceptibility, QSM provides a more precise quantitative estimation of susceptibility sources. T2* relaxometry cannot differentiate between paramagnetic and diamagnetic susceptibilities, as the magnitude of magnetic susceptibility and the resulting field distortion solely influence it. In contrast, QSM provides more detailed information about tissue chemical composition, as it considers both the magnitude and polarity of magnetic susceptibility. For example, calcified tissue and iron-laden tissue may exhibit similar short T2* values, which cannot be distinguished using T2* relaxometry. However, QSM can differentiate them, detecting calcified tissue as a negative susceptibility source and iron-laden tissue as a positive susceptibility source.

Compared to the brain, connective tissues in the MSK system exhibit stronger susceptibility. While white matter and gray matter have susceptibility values of approximately -0.02 ppm and 0.02 ppm, respectively [24], MSK tissues demonstrate susceptibilities several orders of magnitude higher, as shown in Table 1. Moreover, tissues such as tendons, ligaments, and menisci are highly organized, with water molecules tightly bound in the extracellular matrix and macromolecules, resulting in short T2 relaxation times. Combined with their strong susceptibility (i.e., short T2*), these tissues exhibit extremely short T2* relaxation times. Therefore, UTE imaging is advantageous for QSM of MSK tissues, providing enhanced signal-to-noise ratio (SNR) for these short T2* tissues.

Traditional QSM typically uses GRE train imaging, which provides uniformly spaced TEs over several tens of milliseconds within a single acquisition. In contrast, UTE-QSM requires significantly shorter echo spacings to capture multiple images with TEs of less than 1 ms. Due to limitations in gradient hardware and safety concerns related to peripheral nerve stimulation, acquiring multiple GRE images within such a brief period is generally not feasible on most modern clinical MRI systems. Consequently, an interleaved encoding strategy is necessary for UTE-QSM, which may introduce motion between scans. In this study, we demonstrated that this motion is a significant issue, often leading to pronounced streaking artifacts and compromising the quality of the susceptibility map. As shown in Figure 4, appropriate motion correction can effectively reduce these artifacts.

Achieving precise motion registration is challenging, particularly in joints with complex, kinetic movements. Non-rigid motion in soft tissues is complicated to correct. Both uncorrected rigid and non-rigid motions can introduce significant errors in the phase evolution data, leading to pronounced streaking artifacts, particularly in pixels near tissue boundaries with high susceptibility (e.g., around −1.0 ppm for bone and around 0.4 ppm for fat) [14,25] or a strong chemical shift (e.g., −3.5 ppm for fat) [26]. In the in vivo experiment, it was observed that knee QSM is more susceptible to motion effects than ankle QSM, likely due to the knee’s anatomical structure, which makes it more prone to unwanted motion during MRI. The knee joint also contains a greater proportion of soft tissue compared to the ankle joint.

From a clinical perspective, UTE-QSM holds significant potential for improving MSK diagnostics. Its ability to noninvasively and quantitatively distinguish between diamagnetic and paramagnetic sources makes it particularly valuable in assessing conditions characterized by changes in mineralization or iron deposition [16,27,28,29,30,31]. Potential applications include hemophilic arthropathy, osteoarthritis, and osteoporosis, where quantification of hemosiderin [16,17], collagen [32], and bone mineral density [14] may provide useful biomarkers. For example, assessing bone mineral density for cortical and trabecular bone, and differentiating calcified tissues, could be a further potential application of UTE-QSM [30]. UTE-QSM could also detect early degenerative changes in osteoarthritis and subclinical injuries, serving as a biomarker for risk stratification for upcoming severe conditions [25,32]. Similar mechanisms may apply to other hemosiderin-related conditions [16,17], such as tenosynovial giant cell tumor. While these applications highlight the wide-ranging potential of UTE-QSM, they were not the primary scope of this study. Future investigations should specifically focus on these subtopics to further establish the clinical role of UTE-QSM.

In patients with hemophilic arthropathy, where hemosiderin accumulation plays a central role, QSM can detect and quantify iron load more precisely than conventional imaging, aiding in early diagnosis and disease monitoring [16]. Similarly, in osteoarthritis or tendon pathologies, calcifications can be better characterized through susceptibility mapping [25,32], which may potentially guide clinical decisions regarding intervention. Moreover, QSM may serve as a sensitive biomarker for detecting subtle biochemical changes in tissues before gross morphological alterations, providing a tool for earlier detection and longitudinal monitoring. When combined with UTE imaging, which improves visualization of tissues with very short T2* values, the resulting technique expands the diagnostic capabilities of MRI to include previously “invisible” components of the joint, such as tightly bound collagen in tendons and ligaments. UTE imaging is a well-known technique that ranges far beyond MSK imaging [33,34].

In this study, we demonstrated the impact of motion near tissue boundaries. The two-step UTE-QSM significantly improved the resulting susceptibility map by reducing streaking artifacts, as shown in vivo in the knee and ankle joints (Figure 5 and Figure 6, Table 1). This finding supports the idea that strong streaking artifacts arise from abrupt signal changes between different tissues (Figure 4), which complicate signal fitting and contaminate the field map. While the two-step UTE-QSM effectively mitigates streaking artifacts, it does not directly correct for the errors caused by misregistration. Therefore, when combined with more advanced registration methods [35,36], this approach is expected to yield even better results. Additionally, other potential sources of streaking artifacts, such as low SNR and errors in the estimated B0 field map from IDEAL, will be further explored in future studies to enhance UTE-QSM for musculoskeletal imaging.

This study has several limitations. First, only male participants were recruited. While we do not believe this impacts the generalizability of the study, particularly with regard to investigating the effects of motion, it is worth noting that inter-scan motion is sex independent. Second, the study involved a relatively small sample size, with eight participants for knee imaging and seven for ankle imaging. Larger cohorts will enhance statistical power and improve generalizability. This should be done in future studies. Lastly, we did not include a patient cohort (e.g., hemophilia patients with hemophilic arthropathy) [16] to assess the motion effects in lesion detection. Future studies will address these limitations by incorporating a larger number of subjects, including both control and patient populations.

## 5. Conclusions

We showed that inter-scan motion can significantly reduce the quality of UTE-QSM in musculoskeletal imaging. By combining motion registration with a two-step approach that accounts for tissue boundaries, we were able to reduce streaking artifacts and improve image quality. This method increases the reliability of susceptibility measurements and may help UTE-QSM become a more robust tool in clinical practice. Although this study was limited by a relatively small sample size, the results provide a foundation for future research with larger and more diverse groups. UTE-QSM has the potential to support earlier detection and better monitoring of musculoskeletal conditions such as osteoarthritis, tendon injuries, and hemophilic arthropathy.

## Figures and Tables

**Table 1:** Estimated susceptibility values for the knees and ankles of all volunteers. *p*-values less than 0.05 are highlighted in bold.

	ROI	QSM_A_ No Registration (ppm)	QSM_B_ Affine Registration (ppm)	QSM_C_ Affine Registration with Two-Step QSM (ppm)	*p*-Value QSM_A_ vs. QSM_B_	*p*-Value QSM_B_ vs. QSM_C_	*p*-Value QSM_A_ vs. QSM_C_
Knee	Patella Tendon	−0.0138 ± 0.2594	−0.1797 ± 0.1769	−0.1278 ± 0.1322	0.0523	**0.0387**	0.1715
	PCL	−0.5127 ± 0.2582	−0.3835 ± 0.2155	−0.1503 ± 0.1403	0.1572	**0.0026**	**0.0130**
	Meniscus	−0.6010 ± 0.2943	−0.3181 ± 0.1679	−0.1999 ± 0.1134	**0.0467**	**0.0315**	**0.0093**
	Muscle	0.1913 ± 0.0857	0.1334 ± 0.0976	0.0708 ± 0.0933	0.0546	**0.0051**	**0.0003**
Ankle	Achilles Tendon	−0.3660 ± 0.1252	−0.2262 ± 0.1012	−0.2567 ± 0.1145	0.1280	0.1999	0.1860
	Muscle	−0.0853 ± 0.0599	−0.0705 ± 0.0555	−0.0336 ± 0.0285	0.5954	**0.0252**	0.0673

## Data Availability

The original contributions presented in this study are included in the article. Further inquiries can be directed to the corresponding author.

## Abbreviations

UTE	Ultrashort Echo Time
QSM	Quantitative Susceptibility Mapping
MRI	Magnetic Resonance Imaging
TEs	Echo Times
UTE-QSM	UTE-based QSM
RF	Radiofrequency
MSK	Musculoskeletal
IDEAL	Iterative Decomposition of Water and Fat with Echo Asymmetry and Least-squares Estimation
MEDI	Morphology Enabled Dipole Inversion
PCL	Posterior Cruciate Ligament
SNR	Signal-to-Noise Ratio
ROI	Region of Interest
PDF	Projection onto Dipole Fields
